# Explainable artificial intelligence as a reliable annotator of archaeal promoter regions

**DOI:** 10.1038/s41598-023-28571-7

**Published:** 2023-01-31

**Authors:** Gustavo Sganzerla Martinez, Ernesto Perez-Rueda, Aditya Kumar, Sharmilee Sarkar, Scheila de Avila e Silva

**Affiliations:** 1 Programa de Pós-Graduação em Biotecnologia, Universidade de Caxias do Sul, Caxias do Sul, RS Brazil; 2 Unidad Académica de Yucatán, Instituto de Investigaciones en Matemáticas Aplicadas y en Sistemas, Universidad Nacional Autónoma de México, Yucatán, Mérida Mexico; 3 Department of Molecular Biology and Biotechnology, Tezpur University, Tezpur, Assam 784028 India

**Keywords:** Software, Computational biology and bioinformatics, Machine learning

## Abstract

Archaea are a vast and unexplored cellular domain that thrive in a high diversity of environments, having central roles in processes mediating global carbon and nutrient fluxes. For these organisms to balance their metabolism, the appropriate regulation of their gene expression is essential. A key momentum in regulating genes responsible for the life maintenance of archaea is when transcription factor proteins bind to the promoter element. This DNA segment is conserved, which enables its exploration by machine learning techniques. Here, we trained and tested a support vector machine with 3935 known archaeal promoter sequences. All promoter sequences were coded into DNA Duplex Stability. After, we performed a model interpretation task to map the decision pattern of the classification procedure. We also used a dataset of known-promoter sequences for validation. Our results showed that an AT rich region around position − 27 upstream (relative to the start TSS) is the most conserved in the analyzed organisms. In addition, we were able to identify the BRE element (− 33), the PPE (at − 10) and a position at + 3, that provides a more understandable picture of how promoters are organized in all the archaeal organisms. Finally, we used the interpreted model to identify potential promoter sequences of 135 unannotated organisms, delivering regulatory regions annotation of archaea in a scale never accomplished before (https://pcyt.unam.mx/gene-regulation/). We consider that this approach will be useful to understand how gene regulation is achieved in other organisms apart from the already established transcription factor binding sites.

## Introduction

Since archaea have been introduced as a particular domain^1^, much experimentation around these organisms has happened. However, due to their novelty and extreme environment living, there are still open research questions regarding organisms that are important for many global-scale scenarios such as regulation of lighthouse gases, production of biofuels, as well as other bio-industry processes^2,3^. In fact, the extremophile capability of such organisms might allow the exploitation of the severe conditions archaeal proteins are expressed^4^.

When proteins get expressed in a prokaryotic archaeal cell, the DNA-dependent RNA polymerase enzyme (RNAP) transcribes genetic information in an intermediate RNA molecule; this is a conserved process found across all three domains of life^5,6^. For RNAP to carry out RNA synthesis, it firstly needs to be recruited to the correct site in the DNA where protein-coding information is located. What mediates the interaction between RNAP/DNA is the promoter region. This regulatory region is composed of conserved characteristics both in a sequence level^7^ and physico-chemical conformations^3,8^ which has been proposed as characterizers of RNAP binding.

Transcription factor (TF) proteins in an archaeal configuration will assist the recruitment of the RNAP complex to the DNA. These proteins will bind to conserved DNA segments. The main conserved sites up to this day are: (i) a TATA-box, an AT rich area around position − 27 upstream, in which the TATA-box binding protein (TBP) will bind. (ii) two sites located down and upstream the TATA-box in which the two extremities of the Transcription Factor B (TFB) protein will bind and assist RNAP stabilization; and finally, (iii) a conserved area just upstream the Transcription Start Site (TSS) in which the Transcription Factor E (TFE) protein binds to assist the formation of an open complex and allow DNA to be opened and read by RNAP^9,10^.

As more genomic information becomes available, it arises the need for reliable data curation steps to be performed. However, it is costly and laborious to manually annotate this data. Therefore, automated processes of promoter prediction and annotation are appreciated^11^. For that, specific tools have been proposed. Bacterial promoters have been well characterized^12–14^; eukaryotic promoters also count with a consolidated spectrum of tools^15,16^, as per our knowledge, no domain specific tool is available for archaea as the few classifiers that encompass archaeal genomes belong to generic models in prokaryotic organisms, especially bacteria, whose promoters are structurally divergent from archaea.

Many promoter predictors are based upon machine learning (ML). The primary input basis for ML techniques to function upon is through numerical features. In fact, numerical ways to represent genomic information succeeded in characterizing biological and cellular processes such as DNA–protein interaction and DNA melting^3,8,17–19^. To this extent, a well-represented manner to code genetic information is through DNA Duplex Stability (DDS)^20^, in which the free energy released in DNA melting can be quantified. The chemical conformation of different base-pairs interactions is known to yield in differentiated DDS levels.

Many promoter predictors use ML as their form of classification. In this task, different algorithms have been proposed, such as Artificial Neural Networks^13,15,19^, Support Vector Machines (SVM)^11^, Recurrent Neural Networks^16^, among others. However, due to the mathematical complexity, many of these tools will function as black-box classifiers, where one just knows the output associated with given input features. The use of Explainable Artificial Intelligence (XAI) started to gain attention in areas highly controlled by ethical standards, such as the medical sciences due to its ability to grant transparency to models. The novelty proposed by XAI ensures that the magnitude of each input feature gets mapped when assigning labels to a prediction^21,22^.

In this work, we hypothesize machine learning can be exploited to deliver a high-scale annotation upon archaeal promoter sequences. For that, we seek to harness the conserved aspect portrayed by the representation of archaeal promoters through DDS and feed it to classificatory models. The use of XAI will pose a form to tightly control the classification process and grant validity to our findings, allowing us to deliver a set of curated promoter sequences in an unmatched scale.

## Materials and methods

### Train/test promoter sequences

A total of 3935 sequence promoters obtained from available transcriptome map of the organisms *Haloferax volcanii*, *Sulfolobus solfataricus*, and *Thermococcus kodakarensis* (1340, 1021, and 1248 promoters, respectively). Promoters were derived from the transcripts of the organisms. In addition, 405 promoters in-silico predicted^19^ of *Aciduliprofundum boonei* and *Thermofilum pendens* were considered. In brief, Martinez et al. (2022) employed machine learning and statistics to validate promoter sequences of unannotated archaea. We considered a region namely core promoter, which is located from − 80 to + 20 aligned to the Transcription Start Site as this region is known to represent promoter activity^23^.

### Negative control datasets

For each sequence present in the train/test dataset, we generated a shuffled version of it for control purposes. It has been previously benchmarked by Ref.^19^ that among non-coding and shuffled promoter sequences, the shuffled version of a control dataset yielded in the worst classification. Thus, we opted to use this method of obtaining negative sequences to stress our classification model. The dataset containing the train/test data and the control sequences used in this work is publicly available at https://github.com/gustavsganzerla/archaeal_promoters. We opted to maintain a 1:1 proportion in true/false sequences in our train/test data as the binary classification achieved by Support Vector Machines was reported to perform better when classifying balanced datasets^24^.

### Independent promoter sequences for validating the classification method

We selected an independent collection of 2719 experimentally validated promoter sequences from the archaeon *T. Kodakarensis* KOD1 deposited in the Prokaryotic Promoter Database^25^ to assess how our classification model would behave upon independent data.

### Numerical representation of genetic information

There are several physico-chemical conformities upon a DNA sequence that can be represented in numbers^26^. Some of these features were found to converge, conveying the level of contained information^3,27^. In this work, we have selected DNA Duplex Stability (DDS), which has widely been used as a way of representing genetic information^8,13,17,19,28^ as it is dependent on the primary sequence reflected by the number of hydrogen bonds keeping them linked. Each combination of di-nucleotides will favor a DDS measurement (Supplementary Material S1). Each of the $G$ values were obtained through ${\Delta }_{i, i+1}^{0}$ two-nucleotide sliding windows (Eq. 1). The application of Eq. (1) resulted in the promoter, validation, and negative control sequences resulted in a fixed length vector composed of 99 elements. An additional element comprising the label (1 = promoter, 0 = non-promoter) was added. The calculation of DDS was previously reported in Ref.^20^.

1 $$G={\Delta }_{i, i+1}^{0},$$

where $G$ means the DDS variation of ${\Delta }_{i, i+1}^{0}$ a nucleotide and its neighbor.

### Defining the classification rationale

We benchmarked the prediction feasibility of our tenfold cross validated data with distinct classification algorithms, them being Support Vector Machines (SVM), Linear Discriminant Analysis (LDA), Classification and Regression Trees (CART), and K-Nearest Neighbors (KNN). Their implementation took place in R (version 4.1.2) through the script available at https://github.com/gustavsganzerla/archaeal_promoters/blob/main/defining_classification_algorithm.R.

We selected the best performing algorithm (i.e., SVM) to classify archaeal promoter sequences. These classifiers were reported successful in dealing with two classes classification of tabular data in which the input features are numeric vectors^29,30^. To benefit from the sklearn (version 1.1.2) data science library, we reimplemented our classifier in Python (version 3.9.7). To map the input dimension into high-dimensional feature spaces, we considered four kernels in the classification task, namely: rbf, polynomial, linear, and sigmoid. The kernel that presented the highest AUROC, accuracy, precision, recall, and specificity was selected. We maintained the default C and gamma parameters implemented by the svm.svc class in the sklearn library, i.e., scale and 1.0. Each SVM model was trained and tested with promoter sequences in a proportion of 0.9/0.1 (train/test) in a stratified tenfold-cross validation procedure found in the *sklearn.model_selection* package. The metrics accuracy, precision, recall, specificity, receiver operator characteristic (ROC), and area under the curve (AUC) were obtained in each validation fold of each classification model and further used to assess the predictions. All of the performance metrics are found in the *sklearn.metrics* package. The Python code containing the implementation of our classificatory model is available as well as the data preprocessing pipeline is available at https://github.com/gustavsganzerla/archaeal_promoters.

### Explainable artificial intelligence (XAI)

We used Shapley Additive Explanations (SHAP) to provide interpretation to the SVM model^31^ (version 0.41.0). In this, SHAP assigns a score for each input feature (${\phi }_{i}$) that is the contribution of a feature $i$ to a prediction. SHAP performs weights and sums all possible feature combinations. Equation (2) describes the SHAP calculation:

2 $${\phi }_{i}=\sum_{S \subset N \backslash \{i\}}\frac{\left|S\right|!\left(\left|N\right|-\left|S\right|-1\right)!}{\left|N\right|!}\left({f}_{S\cup \left\{i\right\}}\left(x\right)- {f}_{S}\left(x\right)\right),$$

where, $S \subset N \backslash \{i\}$ is the subset of all input features of the model. Then, there is the calculation of the difference of the SHAP value for a model containing an *i* feature ${f}_{S\cup \left\{i\right\}}\left(x\right)$ and a model excluding the feature ${f}_{S}(x)$.

The SHAP approach is defined as a theoretical foundation that may be used to explain any prediction model locally and globally. From this, we employed the *kernel.explainer* method in the SHAP module. The kernel SHAP calculation consists of five steps:

I. Sample all possible combination of input features (*i.e.*, coalitions) in the dataset: ${Z}_{K}^{^{\prime}}\in {\left\{\mathrm{0,1}\right\}}^{M}, K \in \{1,\dots , K\}$. (0 = feature absent, and 1 = feature present in the coalition).
II. The prediction of each ${Z}_{K}^{^{\prime}}$ is obtained with the application of ${Z}_{K}^{^{\prime}}$ to the predictive model.
III. The weight of each ${Z}_{K}^{^{\prime}}$ is computed by the SHAP kernel.
IV. The model is fitted.
V. Return the SHAP coefficients ${\phi }_{i}$.

All coalitions of input features need to be fit to a predictive model, as the factorial aspect in Eq. (2) states. The obtention of SHAP values is represented by ${s}^{n}$, where *s* is the number of promoters and *n* is the number of features. As we want to analyze which sites throughout the promoter region might contribute to its prediction, them being known binding sites of transcription factor protein or not, we opted to preserve all features (*n* = 99, derived from a promoter sequence with 100 nucleotides converted to di-nucleotide DDS) belonging to each promoter. Therefore, the calculation of a SHAP kernel is a computationally expensive task when dealing with large datasets composed of many features and observations. To overcome this, we selected 100 non-repeated random promoters from the input data for having the assigning of their promoter label by the SVM explained. The complete implementation of SHAP is available at (https://github.com/gustavsganzerla/archaeal_promoters).

### Motif discovery

We used the MEME suite^32^ to identify conserved motifs in promoter sequences. The following parameters were used in the tool: (i) 100 nucleotide sequence length which comprises the core promoter region depicted in Ref.^23^; (ii) a zero-order background model generated from the supplied sequences; (iii) zero or one occurrence (of a contributing motif site) per sequence; (iv) 10 distinct motifs were located per organism, and the one that is majorly composed of AT nucleotides around positions − 24 to − 32 were considered as TATA-boxes; and, (v) the width of the motifs varied between 6 and 8 nucleotides to capture TATA-boxes accurately.

### Promoter discovery in unannotated datasets

To validate the predictive model proposed, and to perform an in-silico annotation of these upstream regions and extract putative promoters, we selected upstream regions of 135 archaeal organisms deposited in the database RSAT Prokaryotes^33^. We obtained the region where the core promoter sequence has been reported^23^, between − 80 and 0 (relative to the TSS).

A set of upstream sequences unknown to the SVM were fed into the model using the *predict* function. Then, a *y* index (0 non-promoter or 1, promoter) was attributed to each sequence of the validation dataset. Finally, we appended the index as a new column of the original data and extracted all the ones that were identified as promoters.

## Results

### Characterization of the train/test promoter sequences

Our train/test data considers 3935 promoter sequences from five distinct archaea. First, *H. volcanii*, *S. solfataricus*, and *T. kodakarensis* were chosen because they have transcriptome information published, which enabled their promoters to be extracted. Next, we added promoters from *A. boonei* and *T. pendens* as they have been part of an in-silico annotation process reported by Ref.^19^. The organisms *H. volcanii*, *A. boonei*, and *T. kodakarensis* belong to the Euryarchaeota phylum while *S. solfataricus* and *T. pendens* are crenarchaeota.

To show the diversification of our train/test dataset, we compiled their genomic information (Table 1). The five listed archaea are characterized in terms of their genome AT percentage, which ranges from 33.87% in H. *volcanii* up to 68.68% in *A. boonei*. Next, we have performed a motif discovery search with MEME to locate a consensus representing the TATA-box binding site of each set of promoters. From this, we report that not all promoter sequences present a consensual motif around the TBP binding site.

**Table 1:** Structural information of five distinct archaea.

Organism	Genomic AT percentage (%)	Identified TATA-motif	TATA presence
*A. boonei*	68.68		142/142
*H. volcanii*	33.87		211/1340
*S. solfataricus*	65.52		840/1042
*T. kodakarensis*	49.33		506/1248
*T. pendens*	52.79		189/241

A general characterization of each organism in the train/test dataset is provided. The genomic AT% is accounted for. Also, through MEME (please see “Motif discovery” for details), we identified the TATA motif in the sequences of each archaeon. From the motif returned, we scanned the sequences and checked the proportion of promoters that contain a canonical TATA motif as identified by MEME.

### Support vector machines succeed in classifying archaeal promoter sequences

We classified 3935 tenfold cross validated promoter sequences from varied archaea with distinct algorithms to assess their classificatory feasibility (Table 2). From the evaluation metrics present in Table 2, SVM presents a balanced mean value of its metrics of 84.81% (± 0.58), compared with 76.25% (± 6.52), 82.58 (± 3.21), and 80.05% (± 17.27). Moreover, the higher Kappa (i.e., 0.8) value reported in the SVM model states the agreement between real vs. predicted is more reliable in the SVM model.

**Table 2:** Measuring the feasibility of distinct algorithms to classify archaeal promoters.

Algorithm	Accuracy (%)	Precision (%)	Recall (%)	Specificity (%)	Kappa
Support vector machines	84.67	84.55	84.37	85.68	0.80
Classification and regression trees	72.48	75.12	85.77	71.59	0.57
Linear discriminant analysis	81.73	84.52	78.43	85.64	0.64
K-nearest neighbors	72.54	92	59.32	96.37	0.55

Distinct classification algorithms following a tenfold cross validation of a dataset composed of 3935 promoters and 3935 non-promoters of five distinct archaea were benchmarked.

**Figure 1:**
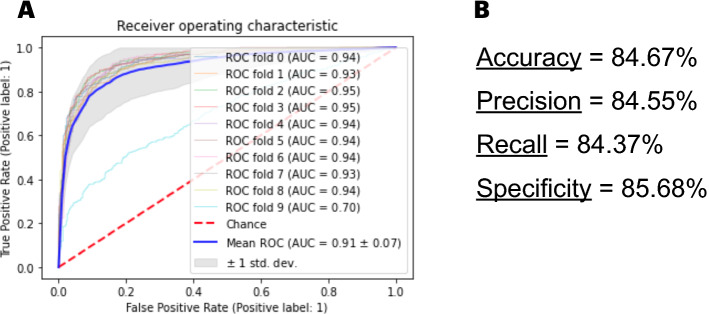
Results of archaeal promoter classification with support vector machines using the rbf kernel. In (**A**) we show the receive operator characteristic curve of a SVM with the RBF kernel in classifying archaeal promoter sequences. A stratified tenfold cross validation procedure was performed to grant statistical validity to the findings. We also calculate the area under the curve (AUC) of each fold, which generated a mean AUC of 0.91 ± 0.07. In (**B**), we show the averaged classification performance of the classificatory model within the metrics accuracy, precision, recall, and specificity for the test dataset. The values displayed are averaged upon the 10 folds of the cross validation.

After selecting SVMs as our classifier, we ran tests using polynomial, radial basis function (RBF), and linear kernels. The kernel that best succeeded in classifying promoter sequences was found to be the RBF. In Fig. 1A, we show the performance metrics of the RBF kernel in classifying stratified tenfold cross validated lists of promoters. The accuracy, precision, recall, and specificity of the model were 84.67%, 84.55%, 84.37%, and 85.68%, respectively. In addition, we have found an AUC score of 0.91 ± 0.07 (Fig. 1B). All the metrics displayed are averaged results of the cross-validation process. Additionally, we have included the ROC curves and performance metrics for the polynomial and linear kernels in Supplementary Material S2.

**Figure 2:**
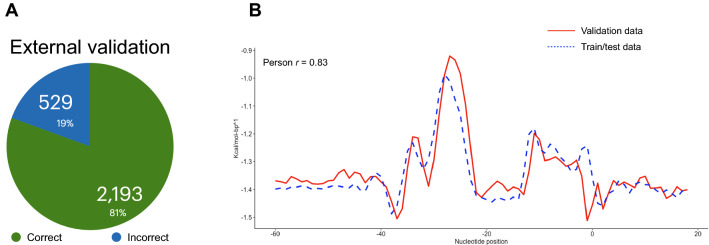
Model validation with external data. In (**A**), we show the rate of our SVM model in assigning the TRUE label to 2719 experimentally validated promoter sequences of the archaeon *Thermococcus kodakarensis*. The sequences are available in the Prokaryotic Promoter Database. In (**B**), we demonstrate the DNA Duplex Stability profile of the 3935 promoter sequences used to train/test the model (dashed blue line) against the 2719 external sequences (continuous red line). The two promoter datasets showed a strong level of correlation (*r* = 0.83).

Moreover, we validated our method with additional 2719 experimentally validated promoter sequences from *T. kodakarensis*. From that, our method correctly labeled 2193 sequences as promoters, totalling 80.65% of precision for identifying true promoters (Fig. 2A). The precision achieved in the test segment of our study (i.e., 84.55%) and the precision obtained with independent data (80.65%) are similar. Additionally, we provide a visualization of the DDS profile of the unseen promoter sequences in Fig. 2B as well as a Pearson correlation (*r* = 0.83) indicating strong correlation between the two datasets comprising of known archaeal promoters.

### Providing interpretability for the SVM model

**Figure 3:**
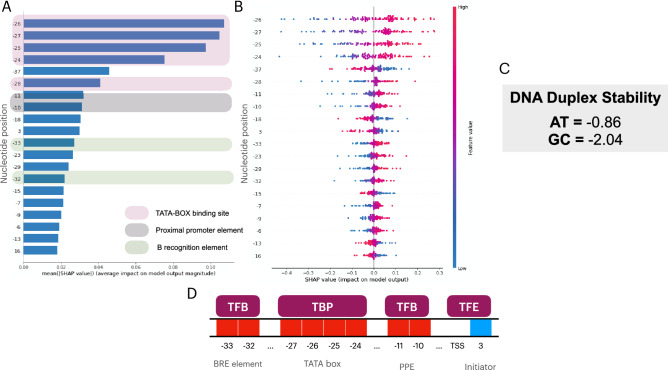
Interpreted SVM model in predicting archaeal promoter sequences. In (**A**), we show the variable importance of the main 20 input features considered by the SVM model in order to classify promoter sequences from archaea. Next, in (**B**), we measured the SHAP impact of each variable in classifying 100 random promoter sequences from the train/test data. The average impact on SHAP (x axis) might be either positive or negative for a specific observation. The fine balance of reds and blues in our model indicated the success of the SVM in labelling these promoters correctly. Therefore, the decision pattern of the model considered a positive SHAP average (i.e., more chances of it being classified as a promoter) for sequences whose input features were either negative or positive, according to the y axis. Next, in (**C**), we averaged the impact on stability of GC (GG, GC, CG, or CC) and AT-composed dinucleotides (AA, AT, TA, AA). Finally, in (**D**), we mapped some of the input features from Fig. 2A,B to match the binding site of TBP, TFB, and TFE proteins. Both panels A and B were outcomes of the SHAP package in Python (version 0.41.0).

To map the decision pattern of our SVM model in classifying promoter sequences and provide global interpretability to our model, we employed SHAP upon 100 random promoters from our train/test data. In Fig. 3A, we show how XAI can improve the promoter prediction. Firstly, we mapped the importance of the nucleotide position to assign the promoter label to a sequence (Fig. 3A). We show that the most discriminatory nucleotide sites that have TF proteins binding are: (i) − 24, − 25, − 26, − 27, − 28, and − 29; (ii) − 32 and − 33; (iii) − 10 and − 11; (iv) + 3. Additionally, we report the positions: − 18, − 37, − 15, − 7, − 6, − 13, and + 16. Next, in Fig. 3B, we show the negative and positive relationship of the predictors with the target variable set to promoters. This shows that the higher a SHAP value (red) in the nucleotide positions − 33, − 32, − 29, − 28, − 27, − 26, − 25, − 24, − 23, − 11, − 10, − 9, − 7, − 6, and the lower the SHAP value in the position − 37, − 18, − 15, − 13, 3, 16, the more likely a sequence is to be labelled as a promoter. Furthermore, in Fig. 3C, we show the high/low values attributed by our structural parameterization in DDS, where GC base pairs tend to contribute more negatively to the energy destabilization of the sequence, while AT tend to be more positively to energy destabilization. Therefore, features where high SHAP values (red) contribute positively to the promoter label are areas in which AT base pairs (higher DDS) are expected.

Next, in Fig. 3D, we show the binding of transcription factor proteins in specific segments of the promoter element. The binding sites of TFB, TBP, and TFE are mapped to the same output obtained in our model explanation step. Finally, we also report some sites downstream the TSS such as the position + 16 and spacers found between TFBS (i.e., positions − 37, − 28, − 18, and − 15) that were considered important for a sequence to be flagged as a promoter.

**Figure 4:**
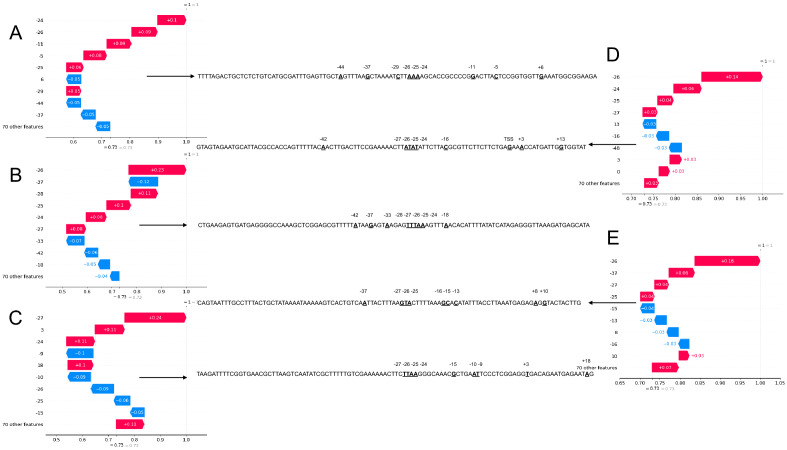
Local interpretation of promoter sequences. We have locally explained the decision pattern of our SVM in assigning the class 1 (i.e., promoter sequence, True Positive) to five random promoters from the train/test dataset. (**A–E**) individually represent a promoter, whose sequence is beside each panel with an arrow indicating it. The 9-most contributing nucleotide positions were selected to be shown. In each sequence, the corresponding nucleotides where the local interpretation assigned either a positive or negative SHAP value in predicting a class 1 are boldfaced and underlined. All panels (**A–E**) were generated with SHAP (version 0.41.0).

Finally, we also used the local interpretability feature enabled by SHAP. To individually explain how the SVM classified instances of promoters, we selected 5 random promoters from our train/test dataset (Fig. 4). The positions − 24, − 25, and − 27 were the sites the mostly identified these five promoter sequences, converging with the globally identified rule for promoter sequences. Moreover, we noted that higher SHAP values for positions − 24, − 25, − 26, − 27, and − 28 contributed to the prediction of a class 1 (i.e., promoter sequence). We previously identified (Fig. 3C) that the more positiveness of a SHAP value is the more predominant its AT content should be.

### Promoter discovery in unannotated upstream regions

We used our SVM model to deliver genomic annotation on archaeal organisms whose promoters have not been yet encountered. For that, we fed the SVM model with 346,174 upstream sequences from 135 archaea. Our predictor returned 85,346 putative promoter sequences. We show in Table 3 an overlook of our prediction, containing the organism’s name, the number of upstream sequences deposited in RSAT, the number of upstream sequences our method indicated to be promoters, the percentage of upstream sequences resulting in predicted promoters, the AT percentage of the upstream sequences of the organism, the conserved TATA motif identified in MEME in a sequence logo format, the nucleotide composition of each motif, and the presence of the motif in the predicted promoters. We noticed the first five archaea with less AT are all members of the halobacterium (*Haloferax* and *Halobacterium*) family and have halophilic characteristics. On the other hand, the organisms with more AT are all members of the methanogen archaea (*Methanothermococcus*, *Methanococcus*, *Methanosarcinia*, and *Methanobrevibacter*). We have opted to display in Table 3, the 5 archaea with most and least promoters predicted by our method (the complete table with all organisms is available in Supplementary Material S4). We also performed a Pearson correlation with number of promoters identified and AT content (Supplementary Material S5) and found that there is a strong correlation (i.e., 0.86) between the variables, i.e., absolute number of promoters identified by our method and genomic AT percentage.

**Table 3:** Characterization of highest and lowest AT containing upstream regions.

Organism	Upstream sequences	Predicted promoters	% predicted as promoters	Genome AT percentage	TATA motif	Presence in predicted promoters (%)	Motif
*H. hubeiense*	3249	424	13.05	36.66		4	WWWAAATA
*N. moolapensis*	2875	394	13.7	38.13		35	DTWTAWT
*H. mukohataei*	3339	483	14.46	38.60		14	TWTWTRTH
*H. salinarium*	2715	434	15.98	37.90		4	AADAATAT
*H. gibbonsii*	3835	632	16.47	37.99		4	AATATBWT
*…*							
*M. ruminantium*	2278	1515	66.50	78.37		100	HWWKDR
*M. stadtmanae*	1589	1090	68.59	79.10		100	HWHHAR
*M. okinawensis*	1679	1217	72.48	80		100	YMWMHW
*M. voltae*	1739	1280	73.60	79.79		100	DKWRRT
*M. olleyae*	1866	1473	78.93	81.54		100	WDHWDD

We show the: (i) five first archaea our model most predicted promoters for, and; (ii) the five last organisms with promoters associated to. We show the number of upstream sequences deposited for each organism in RSAT, the number and percentage of original sequences predicted as promoters, and the AT nucleotide composition. Additionally, we have submitted the identified promoters to the MEME suite to capture the basal TFBSs, a process which returns the TATA element of each organism, the IUPAC motif, upon which we have scanned our sequences to discover their frequencies.

**Figure 5:**
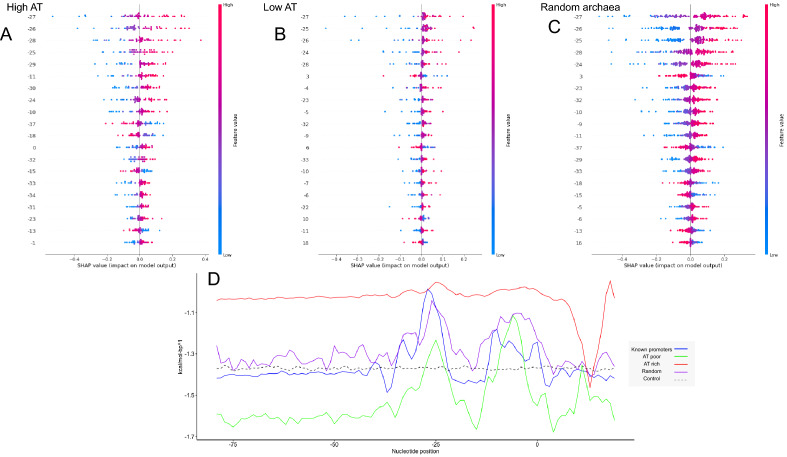
Model interpretation in an SVM discovering new promoters. We show the decision pattern of SVMs in finding new promoter sequences of 135 archaea. In (**A,B**), we show, respectively, how our SVM managed to find promoters in the five AT-most-containing and five AT-least-containing archaea. The most important genomic positions are listed in the y axis from top-down orientation. High SHAP values (red) indicate a predominance of AT nucleotides while low SHAP values (blue) show GC prevalence. In (**C**), we show how our method encountered regulatory regions in a dataset comprising one random promoter from each archaeon. In (**D**), we show a longitudinal DNA Duplex Stability profile for each database employed plus a control set made from shuffled experimentally validated archaeal promoter sequences. Also, in Supplementary Material S4, we include the variable importance for each SHAP plot included in Figure. (**A–C**) were generated with SHAP (version 0.41.0). (**E**) Was generated with the ggplot library (version 3.3.6) in R (version 4.1.2).

To bring evidence that the predicted promoters match the same profile as the experimentally validated ones, we have conducted a new round of SHAP explanations. For that, we have obtained three new datasets: (i) one containing 10 random promoters of each archaeon with more AT genomic content (reported in Figure, Table 3); (ii) a second containing 10 random promoters of each archaeon with less AT content; and (iii) a third containing one random promoter of each one of the 139 archaea in which we proposed to annotate upstream regions. First, in Fig. 5A–C, we show that independently from the AT percentage of the organism, the identification of promoters follows the same global rule: a predominance of AT is scanned around positions − 25 to − 28. Also, the exact site of − 27 upstream the TSS is the most important feature for both types of promoters (i.e., high and low AT). Second, we report that a preference for GC nucleotides in position + 3 was not found in high AT archaeal promoters. In fact, downstream positions were found to be a good characterizer for promoter sequences predicted within non-AT high organisms.

In Fig. 5D, we show in longitudinal terms the profile of four sets of promoters (i.e., the experimentally validated sequences used in train/test, all the predicted promoters from the first 5 AT rich archaea, all the predicted promoters from the first 5 AT poor archaea, and the shuffled sequences as control). We report that all series except the control present a peak in position − 27 and its vicinities, matching the decision pattern of the SVM which considered this area as the most discriminatory one. Therefore, the way that our SVM model assigned a label to an unknown promoter matches the same process performed with experimentally validated regulatory sequences, granting validity to the findings. The lists of predicted promoters are available at https://pcyt.unam.mx/gene-regulation/.

## Discussion

We journey through our results first by showing that even experimentally verified promoters lack canonical TFBS that characterize them in a sequence level, moreover, this is a reductionist approach that does not encompass the general profile of many archaea. For us to achieve that, we made use and extended to quantitative levels already proposed arguments that state the representation of genomic information with numerical features regarding the physicochemical processes within the cell might enlighten genomic annotation^34^. The capability of capturing information in a numerically represented DNA might depict genomic areas that are prone to interaction with external elements such as proteins driving transcription^8,17,35^. We argue that the constant interaction between promoters and TF proteins is driven by properties that transcend the information contained in the primary sequence as our model captures promoters even in organisms with limited AT content and, consequently, lacking canonical TATA motifs.

The presence of a TBP binding site has been extensively investigated as the most conserved element found in promoter regions not only in archaea, but as well as in eukaryotes^36,37^. However, the presence of a TBP binding site is not absolute in all archaea, as we have shown. Thus being, we conclude that promoter identification in archaea must consider other aspects of the DNA molecule rather than sheer consensuses found in a sequence level. On top of that, our results presented alternative conserved areas around the archaeal promoter that might be employed in their characterization, some of them having reference in experimental biology assays and others not. One compelling example of this is the − 10 AT rich area found by our method. Although it has been widely debunked and shown that archaea and bacteria are not closely related evolutionary-wise^7^; in fact, the transcription apparatus in these two prokaryotic branches of life differ a lot^38^. Our results show that there is AT conservation in the − 10 element of archaea that was used by our classification rationale to discriminate promoters. This same region plays a pivotal role in the σ-factor driven attachment of RNAP to promoters^9^. Therefore, our results showed a definition of promoter element in archaea that resembles the other offspring of the prokaryotic branch in the tree of life, suggesting their promoters might share characteristics.

As common as it is with many predictive models, uncertainty is present in ours in a way that about 15% of the train/test sequences were not correctly labelled. Moreover, 19% of our independent dataset was also incorrectly labelled. We attribute the uncertainty of our model due to (i) the simplicity of SVM classification, which favors downstream decision-making processes, as we interpreted our results with XAI; (ii) the ability of SVM classifiers to deal with separable classes^39^ in a way that promoters will likely contain TFBS and make them distinguishable^3^; (iii) the effectivity in SVMs in classifying multidimensional data^40^, as we used the entire namely core promoter region (100 nucleotides) instead of using sites known to contain interaction between DNA and TFs, we went further and used a lengthier input sequence in order to identify genomic areas whose biological properties are not fully consolidated upon the binding of TF proteins, enabling us to explore biological databases in an novel way. Other tools presented a better classificatory performance in predicting regulatory elements, for instance^19^, with archaea^16^ with eukaryote, and Ref.^13^ with bacteria have used Artificial Neural Networks, which is mathematically more complex than our SVMs^41^ as part of classification. However, none of the authors proposed to use XAI to interpret the decision patterns of their classifiers.

By exploring our results, we noticed the predominance of halophilic archaea in low-AT containing organisms. This phenomenon has previously been explored and it was concluded that hypersaline environment drives adaptations that distinguish halophilic prokaryotes. This organisms’ genome benefits from a higher GC content to avoid UV induced thymidine formation, which might happen to organisms inhabiting shallow coastal areas with high UV exposition^42^. In our promoter identification method, even though less promoters have been encountered in halophilic organisms, we showed that we succeeded in locating regulatory regions with promoter-like activity proposed by an in-silico method. The interpretation of our classification rationale was found to be similar in organisms with both high and low AT content. Therefore, we have proposed a global classifier for organisms with varied nature that was able to deliver promoter sequences from archaea at a non-matched scale.

Our study faced limitations towards delivering the final user a webserver in which sequences are inputted and our tool would return their probability of being a promoter. While progress is being made on this, we turned the entire pipeline we developed for this study, including the train/test dataset, publicly available at https://github.com/gustavsganzerla/archaeal_promoters.

We highlight our work in terms of us being able to know the decision patterns of our predictors. XAI has been used to open the black-box that characterizes many AI-based predictions. Health care^43^, pharmacological^44,45^ applications have used XAI to show how algorithms make their decisions, adding transparency to decision-making processes based in AI. Up to this date, no records of XAI being used in regulatory region annotation have been found, which makes our method of classification funded in biological explanations for why and how the classification happened. Therefore, the results gathered in this study will not only deliver the scientific community a novel dataset of in-silico curated archaeal promoters to be explored but new crystal-clear insights on how archaeal gene regulation works.

## Supplementary Information

Supplementary Information 1.

Supplementary Information 2.

Supplementary Information 3.

Supplementary Information 4.

Supplementary Information 5.

## Data Availability

The datasets generated and/or analysed during the current study are available in a public repository https://pcyt.unam.mx/gene-regulation/.
