# Supplementary material for: Explainable artificial intelligence as a reliable annotator of archaeal promoter regions
Source: Sci Rep. 2023 Jan 31;13:1763. doi: 10.1038/s41598-023-28571-7 (PMC9889792; doi:10.1038/s41598-023-28571-7)
Supplement: Supplementary file 1 — Supplementary Information 1. [file 41598_2023_28571_MOESM1_ESM.docx]

**Supplementary Material S1** – DNA Duplex Stability for every possible pair of di-nucleotides.

We provide the DNA Duplex Stability (DDS) value achived by SantaLucia and Hicks (2004) for every possible combination of di-nucleotides. The whole dataset of this study was converted into DDS for genetic information to be represented into numerical attributes.

| **Dinucleotide** | **DDS** |
| --- | --- |
| AA | −1.00 |
| AT | −0.88 |
| AC | −1.45 |
| AG | −1.3 |
| TT | −1 |
| TA | −0.58 |
| TC | −1.28 |
| TG | −1.44 |
| CC | −1.28 |
| CA | −1.45 |
| CT | −1.28 |
| CG | −2.24 |
| GG | −1.84 |
| GA | −1.3 |
| GT | −1.44 |
| GC | −2.24 |
