# Supplementary material for: Explainable artificial intelligence as a reliable annotator of archaeal promoter regions
Source: Sci Rep. 2023 Jan 31;13:1763. doi: 10.1038/s41598-023-28571-7 (PMC9889792; doi:10.1038/s41598-023-28571-7)
Supplement: Supplementary file 2 — Supplementary Information 2. [file 41598_2023_28571_MOESM2_ESM.docx]

**Supplementary Material S2** – Different kernels of support vector machines classifying archaeal promoters.

We show the classification performance of two SVM kernels, i.e., polynomial (**Supplementary Material S2-A**) and linear (**Supplementary Material S2-B**). The performance of each kernel in classifying a stratified 10-fold dataset of 3935 archaeal promoters and 3935 non-promoters was assessed in: *i*) the mean ROC value; *ii*) accuracy; *iii*) precision; *iv*) recall; and *v*) specificity.
