# Supplementary material for: Explainable artificial intelligence as a reliable annotator of archaeal promoter regions
Source: Sci Rep. 2023 Jan 31;13:1763. doi: 10.1038/s41598-023-28571-7 (PMC9889792; doi:10.1038/s41598-023-28571-7)

**Supplementary Material S3** – SHAP explanations of archaeal promoters with varied AT content.

We display the feature importance identified by SHAP on slices of the training dataset. In **Supplementary Material S3-A**, we explained the decision pattern with 10 random promoters from the five archaea with the least genomic AT content. In **Supplementary Material S3-B**, we provide the feature importance of 10 random promoters from the five archaea with the highest genomic AT content. In **Supplementary Material S3-C**, we show the feature importance of 10 random promoters in the whole annotation dataset. In the three instances, independently from the AT content, the position -27 (relative to the TSS) is the most important feature considered to tell apart a promoter.


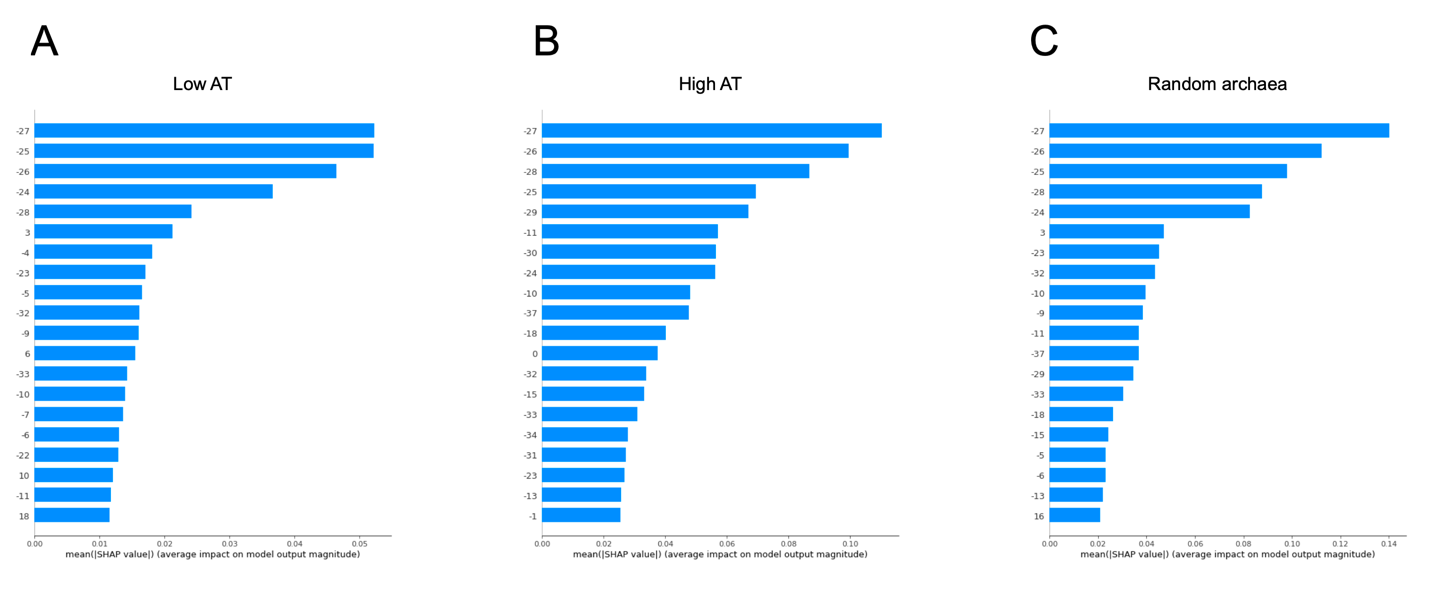

Supplement: Supplementary file 3 — Supplementary Information 3. [file 41598_2023_28571_MOESM3_ESM.docx]
