# Supplementary material for: Explainable artificial intelligence as a reliable annotator of archaeal promoter regions
Source: Sci Rep. 2023 Jan 31;13:1763. doi: 10.1038/s41598-023-28571-7 (PMC9889792; doi:10.1038/s41598-023-28571-7)
Supplement: Supplementary file 4 — Supplementary Information 4. [file 41598_2023_28571_MOESM4_ESM.docx]

**Supplementary Material S4** – Information on organisms whose upstream sequences were annotated into promoters.

We display a table with the number of upstream sequences available; the absolute number of promoter sequences predicted out of upstream sequences by our method; the percentage of total sequences predicted as promoters; and the AT percentage of the upstream sequences.

| **archaeon** | **upstream_sequences** | **predicted_promoters** | **% predicted as promoters** | **AT percentage** |
| --- | --- | --- | --- | --- |
| **Halobacterium_hubeiense_GCF_001488575.1_Halobacterium_hubeiense_JI20-1** | 3249 | 409 | 12.58848877 | 36.66966148 |
| **Halobacterium_salinarum_GCF_000006805.1_ASM680v1** | 2715 | 324 | 11.93370166 | 37.9069581 |
| **Haloferax_gibbonsii_GCF_001190965.1_ASM119096v1** | 3835 | 615 | 16.03650587 | 37.9906331 |
| **Natronomonas_moolapensis_GCF_000591055.1_ASM59105v1** | 2875 | 378 | 13.14782609 | 38.13912337 |
| **Halomicrobium_mukohataei_GCF_000023965.1_ASM2396v1** | 3339 | 464 | 13.89637616 | 38.602647 |
| **Halobiforma_lacisalsi_GCF_000226975.2_ASM22697v3** | 4235 | 577 | 13.62455726 | 39.13351327 |
| **Halopiger_xanaduensis_GCF_000217715.1_ASM21771v1** | 4237 | 602 | 14.20816616 | 39.70018732 |
| **Natronomonas_pharaonis_GCF_000026045.1_ASM2604v1** | 2817 | 353 | 12.53106141 | 39.83183301 |
| **Halodesulfurarchaeum_formicicum_GCF_001886955.1_ASM188695v1** | 2143 | 288 | 13.43910406 | 39.83670755 |
| **Natronococcus_occultus_GCF_000328685.1_ASM32868v1** | 4240 | 581 | 13.70283019 | 39.86191733 |
| **Halorubrum_lacusprofundi_GCF_000022205.1_ASM2220v1** | 3654 | 508 | 13.90257252 | 40.07024975 |
| **Natrinema_pellirubrum_GCF_000230735.2_ASM23073v3** | 4309 | 566 | 13.13529821 | 40.07069232 |
| **Halanaeroarchaeum_sulfurireducens_GCF_001011115.1_ASM101111v1** | 2237 | 336 | 15.02011623 | 40.90997532 |
| **Haloterrigena_turkmenica_GCF_000025325.1_ASM2532v1** | 5225 | 776 | 14.85167464 | 41.04382054 |
| **Halorhabdus_utahensis_GCF_000023945.1_ASM2394v1** | 2994 | 406 | 13.56045424 | 41.26274393 |
| **Halorhabdus_tiamatea_GCF_000470655.1_HATI1** | 3122 | 432 | 13.83728379 | 41.33544201 |
| **Halalkalicoccus_jeotgali_GCF_000196895.1_ASM19689v1** | 3808 | 562 | 14.75840336 | 41.48432465 |
| **Natronobacterium_gregoryi_GCF_000230715.2_ASM23071v3** | 3768 | 584 | 15.49893843 | 42.2756114 |
| **Haloarcula_hispanica_GCF_000223905.1_ASM22390v1** | 3843 | 616 | 16.0291439 | 42.68159848 |
| **Methanoculleus_marisnigri_GCF_000015825.1_ASM1582v1** | 2509 | 426 | 16.97887605 | 43.3246192 |
| **Haloarcula_marismortui_GCF_000011085.1_ASM1108v1** | 4297 | 664 | 15.45264138 | 43.51988761 |
| **Haloterrigena_daqingensis_GCF_001971705.1_ASM197170v1** | 3744 | 550 | 14.69017094 | 43.63765155 |
| **Ignicoccus_hospitalis_GCF_000017945.1_ASM1794v1** | 1508 | 167 | 11.07427056 | 43.66999324 |
| **Halostagnicola_larsenii_GCF_000517625.1_ASM51762v1** | 4021 | 583 | 14.49888088 | 44.18452406 |
| **Methanoculleus_bourgensis_GCF_000304355.2_Mb_MS2** | 2654 | 445 | 16.76714393 | 44.27951017 |
| **Halogeometricum_borinquense_GCF_000172995.2_ASM17299v2** | 3887 | 617 | 15.87342423 | 44.76975837 |
| **Haloferax_mediterranei_GCF_000306765.2_ASM30676v2** | 3882 | 618 | 15.91962906 | 44.88925031 |
| **Pyrolobus_fumarii_GCF_000223395.1_ASM22339v1** | 1967 | 279 | 14.1840366 | 44.91594885 |
| **Methanofollis_liminatans_GCF_000275865.1_ASM27586v1** | 2484 | 466 | 18.76006441 | 45.02619678 |
| **Methanosaeta_harundinacea_GCF_000235565.1_ASM23556v1** | 2495 | 446 | 17.8757515 | 45.09032657 |
| **Thermoproteus_uzoniensis_GCF_000193375.1_ASM19337v1** | 2172 | 603 | 27.76243094 | 46.23312626 |
| **Hyperthermus_butylicus_GCF_000015145.1_ASM1514v1** | 1763 | 336 | 19.05842314 | 46.49620787 |
| **Pyrodictium_delaneyi_GCF_001412615.1_ASM141261v1** | 2080 | 346 | 16.63461538 | 46.89842115 |
| **Methanolinea_tarda_GCF_000235685.2_ASM23568v3** | 2087 | 362 | 17.34547197 | 47.53233944 |
| **Acidilobus_saccharovorans_GCF_000144915.1_ASM14491v1** | 1530 | 255 | 16.66666667 | 47.83064198 |
| **Thermococcus_cleftensis_GCF_000265525.1_ASM26552v1** | 2103 | 333 | 15.83452211 | 49.26777849 |
| **Methanoregula_formicica_GCF_000327485.1_ASM32748v1** | 2892 | 515 | 17.8077455 | 49.59372958 |
| **Thermococcus_nautili_GCF_000585495.1_ASM58549v1** | 2204 | 344 | 15.60798548 | 49.62141783 |
| **Desulfurococcus_mucosus_GCF_000186365.1_ASM18636v1** | 1402 | 366 | 26.10556348 | 49.80218757 |
| **Methanoregula_boonei_GCF_000017625.1_ASM1762v1** | 2605 | 489 | 18.77159309 | 49.90030573 |
| **Methanosphaerula_palustris_GCF_000021965.1_ASM2196v1** | 2850 | 459 | 16.10526316 | 50.55528361 |
| **Thermococcus_gammatolerans_GCF_000022365.1_ASM2236v1** | 2186 | 329 | 15.05032022 | 50.73827872 |
| **Pyrococcus_yayanosii_GCF_000215995.1_ASM21599v1** | 1905 | 315 | 16.53543307 | 51.15840545 |
| **Thermoproteus_tenax_GCF_000253055.1_ASM25305v1** | 2045 | 598 | 29.24205379 | 51.31127083 |
| **Thermococcus_eurythermalis_GCF_000769655.1_ASM76965v1** | 2295 | 386 | 16.81917211 | 51.56640632 |
| **Thermococcus_guaymasensis_GCF_000816105.1_ASM81610v1** | 2126 | 320 | 15.05174036 | 52.34009687 |
| **Geoglobus_ahangari_GCF_001006045.1_ASM100604v1** | 2056 | 524 | 25.48638132 | 52.87818067 |
| **Methanocella_arvoryzae_GCF_000063445.1_ASM6344v1** | 3162 | 675 | 21.34724858 | 53.21721606 |
| **Thermococcus_peptonophilus_GCF_001592435.1_ASM159243v1** | 2076 | 317 | 15.26974952 | 53.33504862 |
| **Thermococcus_onnurineus_GCF_000018365.1_ASM1836v1** | 2022 | 315 | 15.57863501 | 53.39340162 |
| **Nitrososphaera_viennensis_GCF_000698785.1_ASM69878v1** | 2880 | 618 | 21.45833333 | 53.43014487 |
| **Thermococcus_piezophilus_GCF_001647085.1_ASM164708v1** | 2150 | 319 | 14.8372093 | 53.50401149 |
| **Pyrobaculum_islandicum_GCF_000015205.1_ASM1520v1** | 2073 | 568 | 27.39990352 | 55.77266264 |
| **Methanocorpusculum_labreanum_GCF_000015765.1_ASM1576v1** | 1874 | 345 | 18.40981857 | 55.89550073 |
| **Methanocella_conradii_GCF_000251105.1_ASM25110v1** | 2516 | 606 | 24.08585056 | 56.34267469 |
| **Haloquadratum_walsbyi_GCF_000237865.1_ASM23786v1** | 2975 | 518 | 17.41176471 | 56.69067954 |
| **Ignicoccus_islandicus_GCF_001481685.1_ASM148168v1** | 1534 | 262 | 17.07953064 | 56.98621837 |
| **Archaeoglobus_fulgidus_GCF_000008665.1_ASM866v1** | 2480 | 636 | 25.64516129 | 57.0063148 |
| **Methanothermobacter_thermautotrophicus_GCF_000008645.1_ASM864v1** | 1868 | 414 | 22.1627409 | 57.26402957 |
| **Archaeoglobus_veneficus_GCF_000194625.1_ASM19462v1** | 2150 | 543 | 25.25581395 | 57.48259727 |
| **Geoglobus_acetivorans_GCF_000789255.1_ASM78925v1** | 2231 | 592 | 26.53518602 | 57.71597822 |
| **Thermococcus_chitonophagus_GCF_900012635.1_Pyrococcus_chitonophagus_genome_sequence** | 2189 | 406 | 18.54728186 | 57.96195038 |
| **Methanothermobacter_wolfeii_GCF_900095815.1_SIV6** | 1776 | 445 | 25.05630631 | 58.1872957 |
| **Pyrococcus_abyssi_GCF_000195935.2_ASM19593v1** | 1967 | 370 | 18.81037112 | 58.1933745 |
| **Thermofilum_uzonense_GCF_000993805.1_ASM99380v1** | 1695 | 396 | 23.36283186 | 58.27100132 |
| **Pyrococcus_kukulkanii_GCF_001577775.1_ASM157777v1** | 2204 | 406 | 18.42105263 | 58.28068521 |
| **Methanothermobacter_marburgensis_GCF_000145295.1_ASM14529v1** | 1769 | 438 | 24.75975127 | 58.44421585 |
| **Thermosphaera_aggregans_GCF_000092185.1_ASM9218v1** | 1424 | 400 | 28.08988764 | 58.49302894 |
| **Methanospirillum_hungatei_GCF_000013445.1_ASM1344v1** | 3426 | 698 | 20.37361354 | 58.74342193 |
| **Desulfurococcus_amylolyticus_GCF_000020905.1_ASM2090v1** | 1458 | 400 | 27.43484225 | 59.26596545 |
| **Methanolacinia_petrolearia_GCF_000147875.1_ASM14787v1** | 2852 | 612 | 21.45862553 | 59.36637828 |
| **Desulfurococcus_amylolyticus_GCF_000231015.2_ASM23101v3** | 1491 | 431 | 28.90677398 | 59.70488759 |
| **Metallosphaera_sedula_GCF_000016605.1_ASM1660v1** | 2375 | 507 | 21.34736842 | 59.82963657 |
| **Ferroglobus_placidus_GCF_000025505.1_ASM2550v1** | 2574 | 649 | 25.21367521 | 59.91989976 |
| **Thermofilum_adornatus_GCF_000446015.1_ASM44601v1** | 1880 | 440 | 23.40425532 | 60.28918681 |
| **Thermofilum_carboxyditrophus_GCF_000813245.1_ASM81324v1** | 1907 | 424 | 22.2338752 | 60.37508817 |
| **Thermococcus_litoralis_GCF_000246985.2_ASM24698v3** | 2451 | 604 | 24.64300286 | 60.39442509 |
| **Archaeoglobus_profundus_GCF_000025285.1_ASM2528v1** | 1856 | 454 | 24.4612069 | 60.43025536 |
| **Pyrococcus_horikoshii_GCF_000011105.1_ASM1110v1** | 1921 | 412 | 21.44716294 | 60.68915424 |
| **Thermoplasma_acidophilum_GCF_000195915.1_ASM19591v1** | 1588 | 354 | 22.29219144 | 60.84140783 |
| **Palaeococcus_pacificus_GCF_000725425.1_ASM72542v1** | 2015 | 390 | 19.35483871 | 61.14205171 |
| **Methanolobus_psychrophilus_GCF_000306725.1_ASM30672v1** | 2972 | 698 | 23.4858681 | 61.61006482 |
| **Archaeoglobus_sulfaticallidus_GCF_000385565.1_ASM38556v1** | 2265 | 593 | 26.18101545 | 61.67072841 |
| **Pyrococcus_furiosus_GCF_000275605.1_ASM27560v1** | 2131 | 603 | 28.29657438 | 61.71442364 |
| **Thermococcus_barophilus_GCF_000151105.2_ASM15110v2** | 2254 | 467 | 20.71872227 | 61.81134328 |
| **Vulcanisaeta_distributa_GCF_000148385.1_ASM14838v1** | 2503 | 729 | 29.12504994 | 61.89581818 |
| **Caldivirga_maquilingensis_GCF_000018305.1_ASM1830v1** | 2066 | 606 | 29.33204259 | 62.59772966 |
| **Methanococcoides_methylutens_GCF_000970325.1_ASM97032v1** | 2339 | 579 | 24.75416845 | 62.69412959 |
| **Methanosarcina_acetivorans_GCF_000007345.1_ASM734v1** | 4963 | 1133 | 22.82893411 | 62.71716414 |
| **Methanoplanus_limicola_GCF_000243255.1_ASM24325v1** | 3087 | 775 | 25.10528021 | 62.76529658 |
| **Methanosarcina_siciliae_GCF_000970085.1_ASM97008v1** | 4299 | 1025 | 23.84275413 | 62.91273313 |
| **Thermococcus_paralvinellae_GCF_000517445.1_ASM51744v1** | 2145 | 412 | 19.20745921 | 62.92282506 |
| **Methanohalophilus_mahii_GCF_000025865.1_ASM2586v1** | 2047 | 525 | 25.64728872 | 63.37727352 |
| **Thermococcus_sibiricus_GCF_000022545.1_ASM2254v1** | 2016 | 440 | 21.82539683 | 63.41669243 |
| **Methanohalophilus_halophilus_GCF_001889405.1_ASM188940v1** | 2063 | 514 | 24.91517208 | 63.44596669 |
| **Methanosarcina_lacustris_GCF_000970265.1_ASM97026v1** | 3519 | 941 | 26.74055129 | 63.55844959 |
| **Metallosphaera_cuprina_GCF_000204925.1_ASM20492v1** | 1966 | 521 | 26.50050865 | 63.57878108 |
| **Methanomethylovorans_hollandica_GCF_000328665.1_ASM32866v1** | 2630 | 672 | 25.5513308 | 63.62449686 |
| **Methanosarcina_horonobensis_GCF_000970285.1_ASM97028v1** | 4452 | 1200 | 26.9541779 | 63.88084126 |
| **Methanosarcina_mazei_GCF_000007065.1_ASM706v1** | 3505 | 982 | 28.0171184 | 64.14007668 |
| **Vulcanisaeta_moutnovskia_GCF_000190315.1_ASM19031v1** | 2448 | 730 | 29.82026144 | 64.23262975 |
| **Methanosarcina_thermophila_GCF_000969885.1_ASM96988v1** | 2749 | 793 | 28.8468534 | 64.45816088 |
| **Methanococcoides_burtonii_GCF_000013725.1_ASM1372v1** | 2569 | 676 | 26.31374076 | 64.59993299 |
| **Methanosarcina_vacuolata_GCF_000969905.1_ASM96990v1** | 3776 | 1053 | 27.88665254 | 65.50643944 |
| **Thermoplasma_volcanium_GCF_000011185.1_ASM1118v1** | 1627 | 472 | 29.01044868 | 65.56066785 |
| **Methanosarcina_barkeri_GCF_000195895.1_ASM19589v1** | 4060 | 1152 | 28.37438424 | 65.70516258 |
| **Methanosalsum_zhilinae_GCF_000217995.1_ASM21799v1** | 2046 | 663 | 32.40469208 | 66.815569 |
| **Staphylothermus_hellenicus_GCF_000092465.1_ASM9246v1** | 1649 | 641 | 38.87204366 | 66.99410491 |
| **Methanobacterium_formicicum_GCF_000762265.1_ASM76226v1** | 2418 | 834 | 34.49131514 | 67.54834935 |
| **Staphylothermus_marinus_GCF_000015945.1_ASM1594v1** | 1661 | 633 | 38.10957255 | 67.72289142 |
| **Cuniculiplasma_divulgatum_GCF_900090055.1_C.divulgatum_PM4** | 1860 | 617 | 33.17204301 | 68.03892375 |
| **Sulfolobus_acidocaldarius_GCF_000338775.1_ASM33877v1** | 2329 | 745 | 31.98797767 | 68.26700081 |
| **Sulfolobus_islandicus_GCF_000022385.1_ASM2238v1** | 3020 | 888 | 29.40397351 | 68.44301113 |
| **Methanocaldococcus_infernus_GCF_000092305.1_ASM9230v1** | 1502 | 559 | 37.21704394 | 69.40020934 |
| **Acidianus_hospitalis_GCF_000213215.1_ASM21321v1** | 2486 | 810 | 32.58246179 | 69.45126489 |
| **Methanohalobium_evestigatum_GCF_000196655.1_ASM19665v1** | 2394 | 868 | 36.25730994 | 69.50035745 |
| **Methanobacterium_congolense_GCF_900095295.1_MCBB** | 2357 | 939 | 39.83877811 | 69.76458194 |
| **Ferroplasma_acidarmanus_GCF_000152265.2_ASM15226v2** | 1949 | 760 | 38.99435608 | 70.29906068 |
| **Ferroplasma_acidiphilum_GCF_002078355.1_ASM207835v1** | 1838 | 770 | 41.89336235 | 70.3948041 |
| **Methanothermus_fervidus_GCF_000166095.1_ASM16609v1** | 1356 | 530 | 39.08554572 | 70.63854297 |
| **Methanobacterium_lacus_GCF_000191585.1_ASM19158v1** | 2531 | 1105 | 43.65863295 | 71.2717721 |
| **Methanobacterium_paludis_GCF_000214725.1_ASM21472v1** | 2443 | 1103 | 45.14940647 | 71.95356032 |
| **Methanocaldococcus_fervens_GCF_000023985.1_ASM2398v1** | 1638 | 784 | 47.86324786 | 72.56108237 |
| **Methanococcus_maripaludis_GCF_000011585.1_ASM1158v1** | 1776 | 800 | 45.04504505 | 72.73968252 |
| **Picrophilus_torridus_GCF_000008265.1_ASM826v1** | 1615 | 793 | 49.10216718 | 72.8268062 |
| **Methanocaldococcus_jannaschii_GCF_000091665.1_ASM9166v1** | 1864 | 960 | 51.50214592 | 72.88167969 |
| **Methanotorris_igneus_GCF_000214415.1_ASM21441v1** | 1843 | 861 | 46.71730874 | 72.97295847 |
| **Methanocaldococcus_bathoardescens_GCF_000739065.1_ASM73906v1** | 1697 | 856 | 50.44195639 | 73.6043569 |
| **Methanocaldococcus_vulcanius_GCF_000024625.1_ASM2462v1** | 1758 | 875 | 49.77246871 | 73.84984079 |
| **Caldisphaera_lagunensis_GCF_000317795.1_ASM31779v1** | 1556 | 783 | 50.32133676 | 74.07550983 |
| **Methanococcus_vannielii_GCF_000017165.1_ASM1716v1** | 1752 | 902 | 51.48401826 | 74.4813805 |
| **Methanobrevibacter_millerae_GCF_001477655.1_ASM147765v1** | 2316 | 1247 | 53.84283247 | 75.64679199 |
| **Methanobrevibacter_smithii_GCF_000016525.1_ASM1652v1** | 1792 | 908 | 50.66964286 | 75.66940743 |
| **Methanococcus_aeolicus_GCF_000017185.1_ASM1718v1** | 1549 | 953 | 61.52356359 | 77.28694542 |
| **Methanobrevibacter_ruminantium_GCF_000024185.1_ASM2418v1** | 2278 | 1481 | 65.01316945 | 78.37362504 |
| **Methanosphaera_stadtmanae_GCF_000012545.1_ASM1254v1** | 1589 | 1031 | 64.88357458 | 79.10324227 |
| **Methanococcus_voltae_GCF_000006175.1_ASM617v2** | 1739 | 1181 | 67.91259344 | 79.79924932 |
| **Methanothermococcus_okinawensis_GCF_000179575.2_ASM17957v2** | 1679 | 1174 | 69.92257296 | 80.00176699 |
| **Methanobrevibacter_olleyae_GCF_001563245.1_ASM156324v1** | 1866 | 1380 | 73.95498392 | 81.54526229 |
