# Supplementary material for: Explainable artificial intelligence as a reliable annotator of archaeal promoter regions
Source: Sci Rep. 2023 Jan 31;13:1763. doi: 10.1038/s41598-023-28571-7 (PMC9889792; doi:10.1038/s41598-023-28571-7)

Supplementary Material S5 – Correlation between number of promoters identified by our method and AT content of archaeal promoters.

We have conducted a statistical (Pearson) correlation between the AT content of all the 135 archaea and number of sequences predicted as promoters by our method.


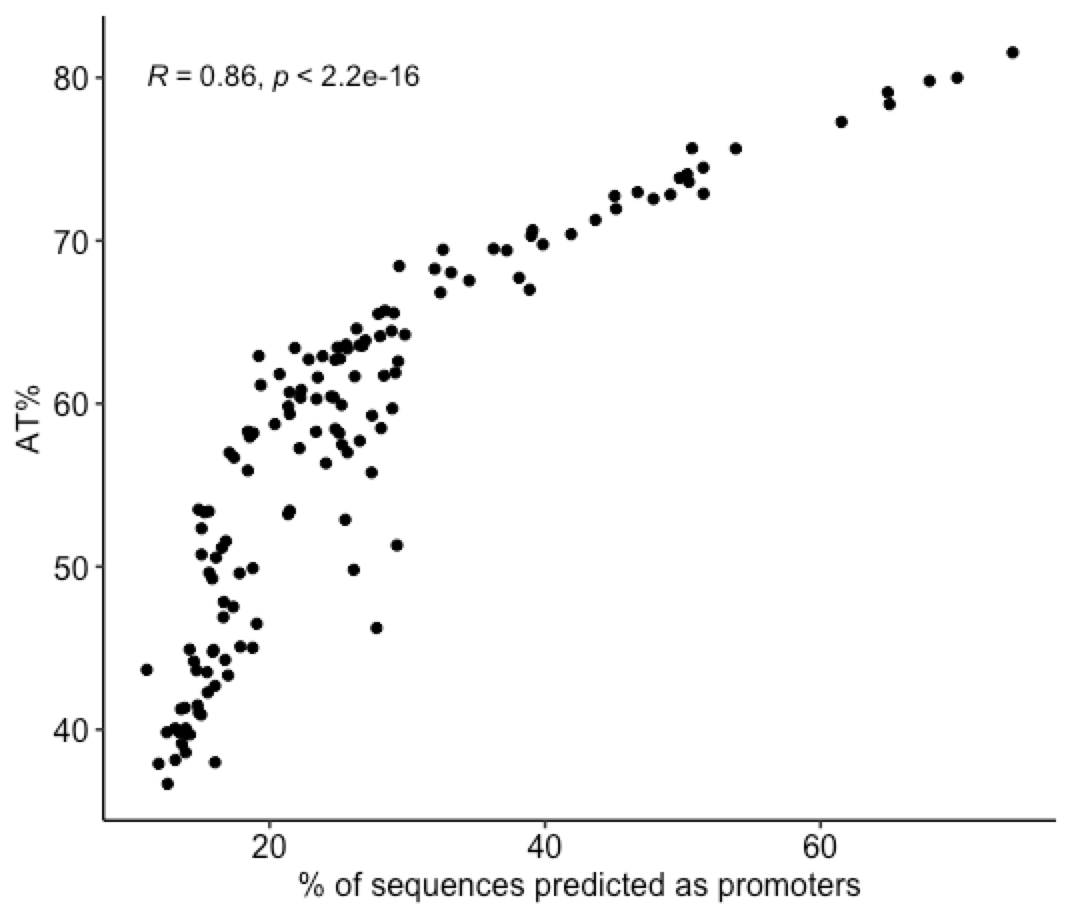

Supplement: Supplementary file 5 — Supplementary Information 5. [file 41598_2023_28571_MOESM5_ESM.docx]
